# O-Glycosylation of snails

**DOI:** 10.1007/s10719-012-9391-4

**Published:** 2012-05-12

**Authors:** Herwig Stepan, Martin Pabst, Friedrich Altmann, Hildegard Geyer, Rudolf Geyer, Erika Staudacher

**Affiliations:** 1 Department of Chemistry; Glycobiology, University of Natural Resources and Life Sciences Vienna, 1190 Vienna, Austria; 2 Institute of Biochemistry, Faculty of Medicine, Justus Liebig University of Giessen, 35392 Giessen, Germany

**Keywords:** Gastropod, Methylated glycans, O-glycosylation, Snail

## Abstract

The glycosylation abilities of snails deserve attention, because snail species serve as intermediate hosts in the developmental cycles of some human and cattle parasites. In analogy to many other host-pathogen relations, the glycosylation of snail proteins may likewise contribute to these host-parasite interactions. Here we present an overview on the O-glycan structures of 8 different snails (land and water snails, with or without shell): *Arion lusitanicus, Achatina fulica, Biomphalaria glabrata, Cepaea hortensis, Clea helena, Helix pomatia, Limax maximus* and *Planorbarius corneus.* The O-glycans were released from the purified snail proteins by β-elimination. Further analysis was carried out by liquid chromatography coupled to electrospray ionization mass spectrometry and – for the main structures – by gas chromatography/mass spectrometry. Snail O-glycans are built from the four monosaccharide constituents: *N*-acetylgalactosamine, galactose, mannose and fucose. An additional modification is a methylation of the hexoses. The common trisaccharide core structure was determined in *Arion lusitanicus* to be *N*-acetylgalactosamine linked to the protein elongated by two 4-*O*-methylated galactose residues. Further elongations by methylated and unmethylated galactose and mannose residues and/or fucose are present. The typical snail O-glycan structures are different to those so far described. Similar to snail N-glycan structures they display methylated hexose residues.

## Introduction

O-Glycans have been found to play an important role in protein stability and tertiary structure, stabilize protein conformation, modulate the activity of enzymes (*e.g*. by reversible attachment of O-linked GlcNAc to cytoplasmic and nuclear proteins) and signaling molecules, are essential for a number of recognition processes and are sorting determinants guiding the modified protein in the cell from the place of biosynthesis to its target location [1–3]. Unlike the N-glycans they may be linked to several different amino acid residues (very often serine or threonine, but also hydroxylysine, tyrosine or hydroxyproline) of the protein and their occurrence cannot be predicted easily by a common consensus sequence [4]. Several monosaccharides have been identified as connection sugar residues to the peptide chain. Extended GalNAc residues linked to serine or threonine residues (mucin-type glycans) are the most frequent and best investigated O-glycans in higher eukaryotes. Eight core subtype formations showing cell-tissue specific patterns related to their function have been described (for a review see [5]). Also GlcNAc-β-Ser/Thr without any elongation, a typical feature for nuclear and cytoskeletal proteins existing in a dynamic equilibrium with phosphorylation [6], and Fuc-α-Ser/Thr as well as Glc-β-Ser, primarily found in epidermal growth factor domains, have been found in eukaryotes [1]. A linkage via mannose has been found to be the main O-glycan type in yeasts, but also some mammalian tissues carry this structural feature, which seems to be relevant in some muscular dystrophies [7], while O-xylosylation is the starting point for the biosynthesis of chondroitin and heparan sulfates, which have roles in development and morphogenesis. Insects show similar O-glycans, however, in more simple versions [8]. The O-glycans of nematodes are rather complex with methylation as further modification [9, 10]. *Xenopus* eggs have O-glycans with up to 10 monosaccharide constituents, often highly fucosylated, with a linkage to the protein via GalNAc [11, 12]. Even parasites (helminths and *T. cruzi*) display small GalNAc linked O-glycans [13]. In plants O-glycans occur as arabinogalactans linked to hydroxyproline or serine (Gal-β-Hyp/Ser), or hydroxyproline is glycosylated with short arabinofuranosides (Ara-α-Hyp, Ara-β-Hyp) [4]. Here we present for the first time an overview on O-glycans of some species of the mollusk branch of the evolutionary tree.

The glycosylation potential of snails is important in general, because some of them are intermediate hosts of pathogens (*Biomphalaria glabrata* and *Lymnaea stagnalis*) or a pest in agriculture. A broad knowledge of their glycosylation abilities may show targets for pest control in the near future. In the course of the years, N-glycan patterns of several snail species have been presented [14–17]. Methylated mannose and galactose residues were found to be frequent constituents.

In the present study we determine the main O-glycan structures in snails and characterize a common core-trisaccharide.

## Material and methods

### Materials and reagents

*Cepaea hortensis, Planorbarius corneus, Arion lusitanicus, Limax maximus* and *Helix pomatia* were collected by the authors under the supervision of Dr. Manfred Pintar (Department of Integrative Biology and Biodiversity Research, Institute of Zoology, University of Natural Resources and Life Sciences, Vienna) in the vicinity of Vienna. *Achatina fulica* and *Biomphalaria glabrata* were bred in the laboratory at 25 °C. *Clea helena* was bought in an aquaristic shop in Vienna. All animals were frozen at −80 °C immediately after collection. All chemicals purchased were of the highest quality available from Sigma or Fluka.

### Preparation of proteins from snail origin for GC-MS analysis

While water living snails could be dissected right away, land living snails were extensively washed to remove the extraneous mucous components. Snails were dissected as previously described [17] and proteins were purified according to [18] with minor modifications: 1 g of wet tissue was homogenised in 10 mL of CHAPS-based lysis buffer (0.5 % (w/v) CHAPS, 150 mM NaCl, 20 mM Tris/HCl, 2.5 mM sodium pyrophosphate, 1 mM ethylene-glycol-bis(2-aminoethylether)-*N*,*N*,*N*′,*N*′-tetraacetic acid and 1 mM EDTA, pH 7.5) and incubated for 72 h at 4 °C. Snail proteins were then reduced by the addition of 500 μL of 10 mM dithiothreitol and incubation at 56 °C for 45 min. Then 500 μL of 55 mM iodoacetamide were added and incubated for 30 min at room temperature in the dark. The samples were dialysed against water over night. The aqueous samples were precipitated with a 4-fold amount of pre-chilled acetone at −20 °C for at least 4 h and then centrifuged at 32000 × g for 60 min. The pellets were resuspended in 0.1 M sodium citrate buffer, pH 4.6, and incubated with 2 units of α-glucosidase from rice [EC 3.2.1.20] at 37 °C for 16 h. Sugars and polysaccharides were eluted from cation exchange chromatography (AG 50 W-*X*2, Biorad, CA) with 2 % of acetic acid, while proteins were eluted by 1.0 M ammonium acetate, pH 9.0. The protein fraction was again precipitated by acetone followed by a similar precipitation by methanol. The precipitates where dried at room temperature.

### Release and purification of O-glycans in preparative amounts

β-Elimination was carried out according to [19]. In short, the dry sample was dissolved in 0.1 M NaOH containing 0.8 M NaBH_3_ and incubated at 37 °C for 68 h. The sample was carefully neutralised by the addition of 2 M acetic acid, dried in a vacuum centrifuge and washed at least twice with 30 % (v/v) methanol. The glycans were directly extracted by solid phase extraction on a porous graphitized carbon cartridge (Supelclean Envi-Carb, 100 mg bed weight, 1 mL column volume or 500 mg bed weight, 6 mL column volume, Sigma-Aldrich, Germany) according to [20] by using ammonium formate buffer/acetonitrile for elution [21] and then on a reversed-phase C18 cartridge (Strata, C18-E, 55 μm, 50 mg bed volume, 1 mL column volume, Phenomenex, Germany) using 5 % (v/v) acetic acid as loading solvent and 25 % (v/v) isopropanol in 5 % (v/v) acetic acid for elution.

HPLC separation was carried out on a porous graphitized carbon (PGC) column (Hypersil-Keystone, Hypercarb 5 μ 150 × 3 mm, Thermo Scientific, Austria) at a flow rate of 0.6 mL/min using 0.3 % (v/v) formic acid adjusted to pH 3.1 with ammonium as solvent A and 95 % (v/v) acetonitrile in solvent A as solvent B. The elution protocol consisted of 2 min at 100 % (v/v) of solvent A followed by a gradient of 28 min till 25 % (v/v) of solvent B and a cleaning step of 4 min at 60 % (v/v) of solvent B. Each fraction was tested by ESI-MS for its glycan content. Single glycan structures were pooled and lyophilised.

### GC-MS-analysis

For monosaccharide analysis alditol acetates and for linkage analysis partially methylated alditol acetates were prepared according to [22]. GC analysis was carried out on a VF 5 ms capillary column (60 m, 0.25 mm inner diameter; 0.1 mm film thickness; Varian, Darmstadt, Germany) and electron impact (EI) mass spectrometry was performed in the positive ion mode using a Polaris Q instrument (ThermoQuest-Analytical Systems) [23].

### Preparation of glycans for PGC-LC-ESI-MS analysis

For analysis of small sample amounts with ESI-MS an in-gel release method for glycans bound to Coomassie stained proteins isolated by SDS-PAGE was used [24, 25]. Reduced oligosaccharides were analyzed by PGC-LC-ESI-MS on a Hypercarb column (0.32 × 150 mm, Thermo Fisher Scientific, Austria) coupled to an Ultimate 3000 (Dionex) capillary HPLC and a Q-TOF Ultima MS (Waters) as described previously [21, 26]. The interpretation of the MS data was performed with the help of the software tool GlycoWorkbench [27].

## Results

### Purification of snail O-glycans for GC-MS

*Arion lusitanicus* proteins were isolated and separated from contaminants through several purification steps in order to obtain sufficient amounts (1–2 μg) of pure glycans for further GC-MS analysis (total monosaccharide and linkage analysis). Prior degradation and separation of storage glycans were essential for further purification success. The preparation of O-glycans was carried out via β-elimination followed by further separation steps. As a final procedure the glycans were separated on a PGC (porous graphitized carbon) HPLC-column by collecting 1-minute fractions which were screened by ESI-MS to gain clean single structures.

### Elucidation of a common core structure for all snail O-glycans

**Fig. 1:**
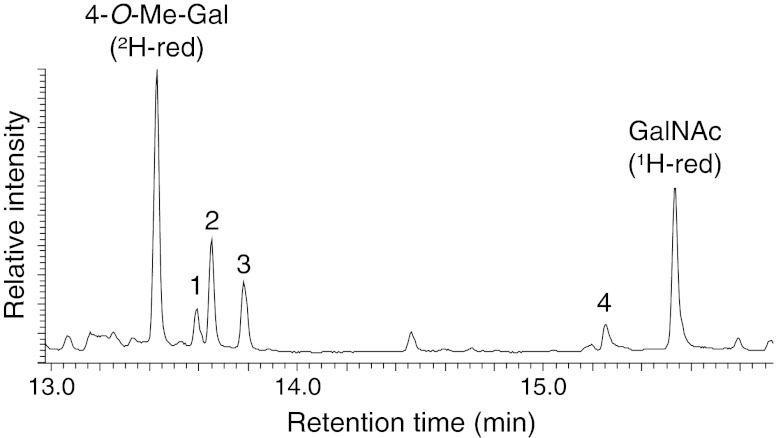
Monosaccharide constituent analysis of the core trisaccharide of *A. lusitanicus* released by [^1^H] reductive β-elimination. Alditol acetates obtained after acid hydrolysis, reduction with sodium borodeuteride and peracetylation or hydrolysis and peracetylation of already existing alditols were identified by GC-MS. Peaks 1–4 arise from contaminating N-glycans and storage oligosaccharides that could not be completely removed (1: Man, ²H-reduced, 2: Glc, ²H-reduced, 3: Gal, ²H-reduced, 4: GlcNAc, ²H-reduced)

**Fig 2:**
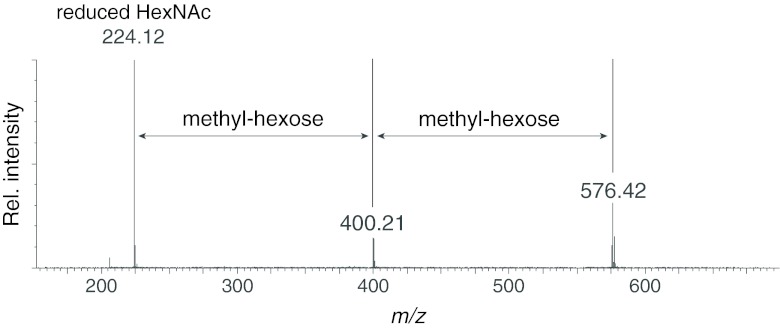
LC-ES-MS/MS spectrum of the core trisaccharide of *A. lusitanicus.* Registered ions represent proton adducts [M+H]^+^

In the course of the β-elimination releasing the glycan from the protein backbone, sodium borohydride was used. In contrast, for reduction of monosaccharides before GC-MS analysis sodium borodeuteride was employed, which allowed further the discrimination between the sugar released by β-elimination and those linked to other sugars previously. In Fig. 1 monosaccharide constituents of the core trisaccharide representing the main structure found in *A. lusitanicus*, with a pseudomolecular mass ([M+H]^+^) of *m/z* 576.3 Da, were determined by GC-MS of the corresponding alditol acetates and identified on the basis of retention times in GC and their characteristic electron impact (EI) mass spectra. The results revealed the presence of a GalNAc-residue which was ^1^H-reduced, while all the other sugars were ^2^H reduced. Therefore, GalNAc can be considered as the protein bound sugar. The high amount of 4-*O*-Me-Gal indicated that this residue is the one which is mainly involved in the elongation of the glycan. Minor compounds were identified as contaminations by N-glycans (GlcNAc and Man) or storage glycans (Glc and Gal). The LC-ESI-MS/MS fragmentation pattern confirmed the occurrence of two methylated hexoses linked to the amino sugar (Fig. 2).

**Fig 3:**
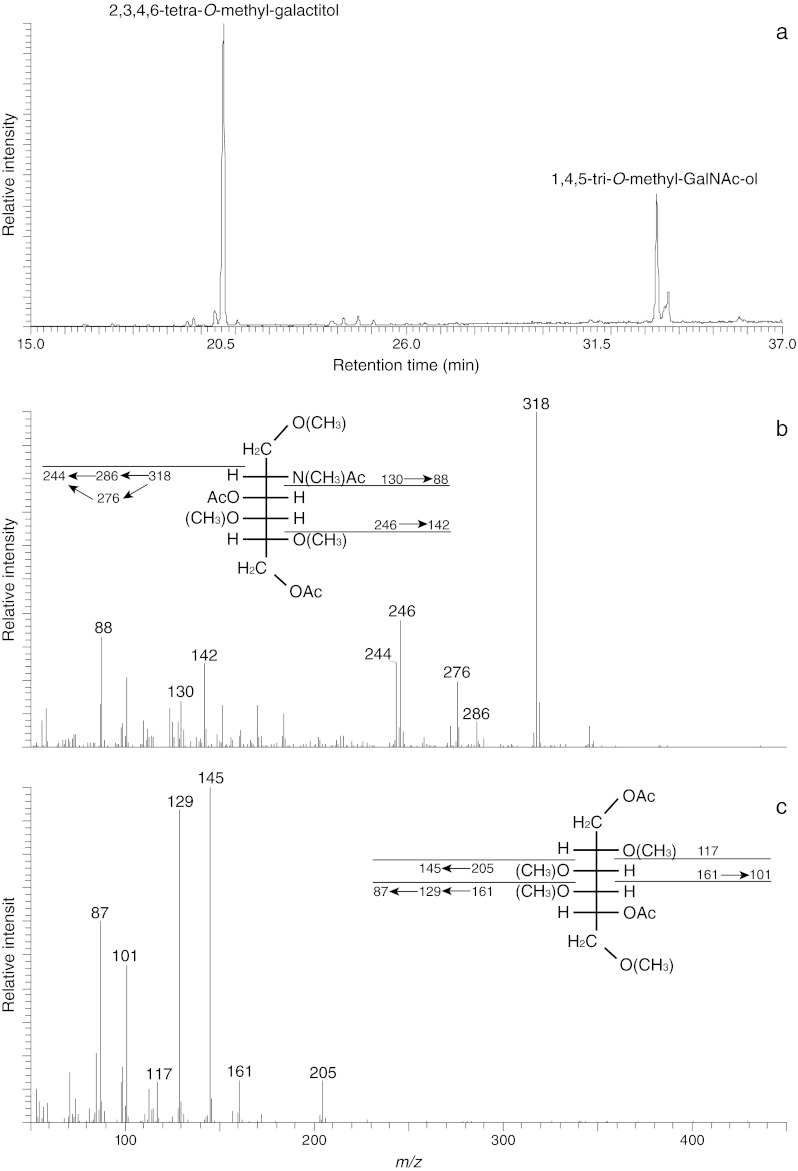
Linkage analysis of the core trisaccharide-alditol from *A. lusitanicus* (**a**) selected ion chromatogram (*m/z* 145 and *m/z* 318) of partially methylated alditol acetates obtained after permethylation with [^1^H] methyliodide, hydrolysis, reduction with sodium borohydride and peracetylation; (**b**) EI-MS spectrum and fragmentation pattern of 1,4,5-tri-*O*-methyl-GalNAc-ol and (**c**) 2,3,4,6-tetra-*O*-methyl-galacitol. Characteristic primary selected secondary fragment ions are assigned

**Fig 4:**
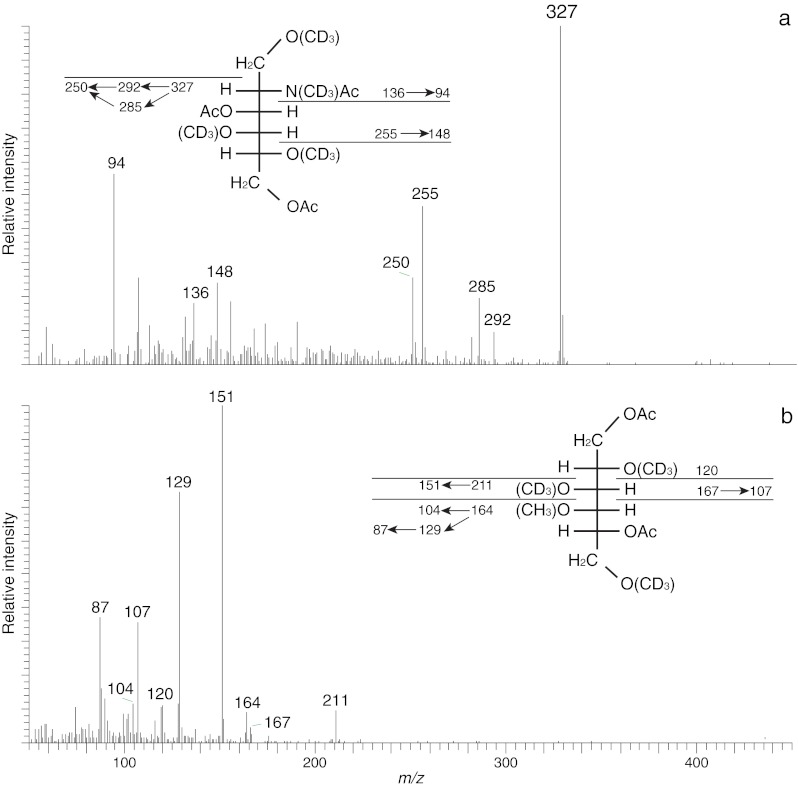
EI-MS spectra and fragmentation patterns of (**a**) 1,4,5-tri-*O*-deuteromethyl-GalNAc-ol and (**b**) terminal 4-*O*-methyl-2,3,6-tri-*O*-deuteromethyl-galacitol obtained after permethylation of the core trisaccharide alditol from *A. lusitanicus* with [^2^H] methyliodide. Characteristic primary and selected secondary fragment ions are assigned

**Fig 5:**
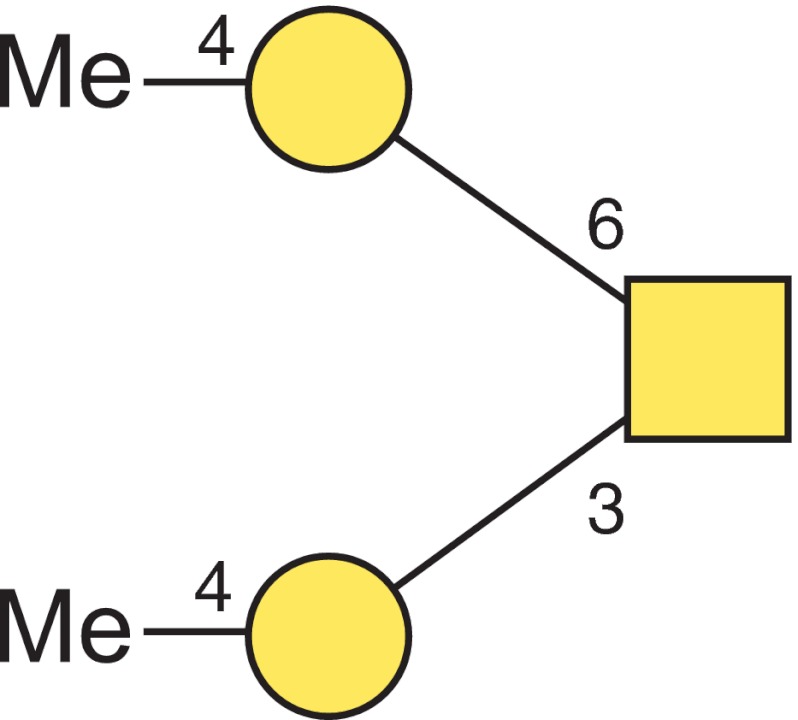
Structure of the core trisaccharide. The structure plot is generated in the notation of the Consortium for Functional Glycomics (http://www.functionalglycomics.org) using the visual editor of “GlycoWorkbench”. This software application is developed and available as part of the EUROCarbDB project (http://www.eurocarb.db.org/applications/ms-tools). Square = GalNAc, Circles = Gal, Me = methyl group

Moreover, GC-MS analysis of partially methylated alditol acetates was carried out for further confirmation of the compounds and also for linkage determination. Methylated sugar standards purified by preparative GC were used to elucidate the type of methylated hexose (Man or Gal) based on retention time. The selected ion chromatogram of the monosaccharide derivatives obtained from the O-glycan core structure identified 2,3,4,6-tetra-*O*-methyl-galacitol at 20.66 min and 1,4,5-tri-*O*-methyl GalNAc-ol at 33.33 min (Fig. 3a) which could be verified by EI-MS data (Figs. 3b and c). To determine the position of the naturally occurring methyl group on the galactose a further permethylation analysis was performed using deuterated iodomethane. This allows in the EI-MS fragmentation pattern the discrimination between the naturally occurring and introduced methylgroups in the fragments by a mass difference of 3 Da. That way the occurrence of terminal 4-*O*-methylated galactose could be confirmed (Fig. 4). Combining these data, the main O-glycan structure of *A. lusitanicus* was elucidated as a trisaccharide containing two terminal 4-*O*-methylated galactoses which are 3- and 6-linked to an inner GalNAc residue (Fig. 5).

**Fig 6:**
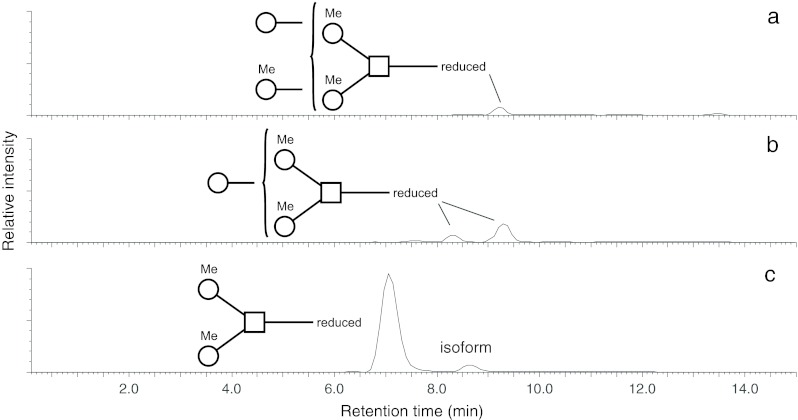
Selected ion chromatograms of partially methylated structures obtained by LC-ESI-MS. **a** Core trisaccharide carrying in addition one methylated and one unmethylated hexose (*m/z* 914.4 [M+H]^+^); **b** Core trisaccharide with one additional hexose (*m/z* 738.3 [M+H]^+^); **c** Core trisaccharide and its isoform (*m/z* 576.3 [M+H]^+^). Square = amino sugar, circle = hexose, Me = methyl group

A small amount of an isoform with the same molecular weight, but slightly later retention time in the PGC (porous graphitized carbon) selected ion chromatogram (Fig. 6c), was identified analogously by GC-MS and EI-MS as trisaccharide containing a GalNAc residue substituted by one 4-*O*-methylated galactose and one 3-*O*-methylated mannose (data not shown).

### Elongated structures

Besides the trisaccharide core several other glycan species were found during purification on PGC-column. They represent mainly elongated versions of the trisaccharide core (Fig. 6a and b). A glycan consisting of the methylated core trisaccharide and one further hexose (*m/z* 738.3 [M+H]^+^) was found. This additional hexose was an unmethylated Gal residue linked alternatively to one of the two arms of the trisaccharide explaining the two isoforms detected by PGC selected ion chromatogram (Fig. 6b). The fourth structure (*m/z* 914.4 [M+H]^+^) which was obtained in sufficient amounts for GC-MS consisted of the core trisaccharide elongated by an unmethylated hexose on one arm and a methylated hexose on the other one (Fig. 6a). The presence of significant amounts of Man and 3-*O*-Me-Man indicated that these two residues were responsible for the elongation.

### Purification of snail O-glycans for LC-ESI/MS

GC-MS analysis using different derivatisation protocols requested a high amount of material and high quality of purification. Especially for snail tissues this was a challenge and means the effort for sample preparation was high in terms of time and material. Therefore this method could not be carried out for a large number of samples in due time. For LC-ESI-MS the requested amounts of glycans were significantly lower. An in-gel β-elimination after SDS-PAGE followed by a short clean-up (PGC-cartridge) procedure was sufficient [25]. The shortened protocol also ensured that no weakly bound modifications, such as sulfate or sialic acids for example, got lost.

### Snail O-glycan overview

Combining our data on snail monosaccharide analysis [28], data from previous GC-MS methylation analysis [23] and the fragmentation results of LC-ESI-MS/MS analysis of selected snails we could determine the main structures of snail O-glycans and classify them according to their modification of the established core structure into six groups: (I) glycans smaller than the core, missing one or both methyl groups or missing one hexose; (II) the trisaccharide core containing GalNAc and two 4-O-methylated hexose residues; (III) the core with additional methylated hexoses; (IV) the core with one or two additional unmethylated hexoses and sometimes one more methylated hexose; (V) glycans containing fucose; (VI) glycans containing one HexNAc and up to six unmethylated hexoses. For an overview on the structures see Table 1.

**Table 1:** Overview of O-glycan distribution in selected snail species given in [%] of total O-glycans. The major glycan structure of each snail is printed in bold. Compositions are given in terms of hexose (Hex), methylated hexose (MeHex), *N*-acetylhexosamine (HexNAc) and deoxyhexose (fucose; dHex)

Glycan composition	Group	Observed mass [M+H]^+^	Calculated mass [M+H]^+^	*Arion lusitanicus*	*Achatina fulica*	*Cepaea hortensis*	*Planorbarius corenus*	*Biomphalaria glabrata*	*Helix pomatia*	*Limax maximus*	*Clea helena*
HexHexNAc	(I) - glycans smaller than the core	386.2	386.17	0	7	0	0	0	0	0	0
MeHexHexNAc		400.2	400.18	5	0	3	1	0	2	0	0
Hex_2_HexNAc		548.2	548.22	traces	0	0	0	traces	0	0	0
HexMeHexHexNAc		562.3	562.23	9	0	0	12	18	26	0	0
(MeHex)_2_HexNAc	(II) - core	576.3	576.25	**54**	**69**	26	12	29	**29**	**100**	**89**
(MeHex)_3_HexNAc	(III) - core with additional methyl hexoses	752.3	752.32	5	2	12	3	0	27	0	0
(MeHex)_4_HexNAc		928.4	928.39	traces	0	0	0	0	0	0	0
(MeHex)_5_HexNAc		1104.4	1104.46	traces	0	0	0	0	0	0	0
Hex(MeHex)_2_HexNAc	(IV) – core with additional hexoses and methyl hexoses	738.3	738.30	14	16	17	16	**45**	12	0	11
Hex_2_(MeHex)_2_HexNAc		900.4	900.36	2	0	**37**	2	0	1	0	0
Hex(MeHex)_3_HexNAc		914.4	914.37	2	5	5	0	5	4	0	0
(MeHex)_2_HexNAcdHex	(V) - fucosylated structures	722.3	722.31	2	2	0	traces	1	traces	0	0
(MeHex)_3_HexNAcdHex		898.4	898.38	6	0	0	0	0	0	0	0
(MeHex)_4_HexNAcdHex		1074.4	1074.44	traces	0	0	0	0	0	0	0
Hex_3_HexNAc	(VI) – HexNAc and hexoses	710.3	710.27	0	0	0	6	1	traces	0	0
Hex_4_HexNAc		872.3	872.32	0	0	0	**46**	0	0	0	0
Hex_5_HexNAc		1034.3	1034.38	0	0	0	3	0	0	0	0
Hex_6_HexNAc		1197.0	1196.43	0	0	0	traces	0	0	0	0

Analyzing snails in detail, it was obvious that the O-glycan trisaccharide core is an important decoration of snail proteins in all investigated species. In five of the analyzed snails (*A. lusitanicus, A. fulica, H. pomatia, L. maximus, C. helena*) this glycan was the most abundant type of O-glycan. *B. glabrata* and *C. hortensis* displayed as their main glycan the trisaccharide elongated by one or two unmethylated hexoses, respectively. Only *P. corneus* was an exception possessing a large amount of Hex_4_HexNAc glycan. Whereas the addition of one hexose to the core was relatively frequent, larger structures were minor compounds or were even not present in several species.

The number of involved monosaccharides in snail O-glycosylation was relatively low. The only detected amino sugar was GalNAc which provided in all cases the connection to the protein. No amino sugars were involved in elongations. Gal and Man were present in unmethylated or mono-methylated (3-*O*-Me-Gal, 4-*O*-Me-Gal, 3-*O*-Me-Man) versions. Fucose, which had been identified as such in previous works [23, 28], occurred only in low amounts and was detected linked to GalNAc as well as to terminal sugar residues by MS/MS fragmentation. No other monosaccharides or any negative charges have been detected in the investigated species.

## Discussion and conclusion

During the last decades our knowledge on glycosylation processes - structure of the glycans and the corresponding biochemical pathways including the responsible enzymes - increased enormously, especially for glycans of mammalian origin. The glycosylation capabilities of other species were only under investigation if their glycans were for any reason connected with human life (*e.g*. some recognition processes of pathogens or allergy on food or plant glycans) or if they were potent candidates for cell culture systems for the expression of therapeutics (some insect, yeast and plant cells). In the meantime more and more invertebrate systems are investigated because of their potential usefulness for biotechnological reasons or to get some deeper insights into the processing pathways of unusual glycan structures. Previous studies on various invertebrates showed that glycosylation has a lot more varieties than we expected as long as only mammals were investigated, but the data are scattered. Still the main focus is laid on N-glycans but the interest in O-glycans is growing.

A number of difficulties arose during snail glycan analysis due to the tough tissue, which was not easy to homogenize and the rigid mucus, which had to be discarded diligently. The removal of storage carbohydrates, which might influence monosaccharide analysis and the separation of N- and O-glycans were other struggles to resolve. While the first was done by α-glucosidase digestion from rice, the latter was an even more difficult task. N-glycosidases (PNGase A and F) did not remove the N-glycans completely and even mild β-elimination conditions seemed to be able to release, at least in part, residual N-glycans which resulted in N-glycan impurities in O-glycan fractions. Furthermore some N-glycans of snails and digested storage glycans are so small (4–5 sugar residues [16, 17]) that they may co-migrate on HPLC with O-glycans. Therefore only a limited number of glycans could be prepared in terms of sufficient amount and purity for permethylation followed by MALDI-TOF-MS and GC-MS linkage analysis. However, in combination with LC-ESI-MS of native glycans which is capable of a high throughput of samples the O-glycan pattern of 8 snail species (land snails with shell: *Achatina fulica, Cepaea hortensis* and *Helix pomatia;* slugs: *Arion lusitanicus* and *Limax maximus;* water snails: *Biomphalaria glabrata, Clea helena,* and *Planorbarius corneus*) could be identified.

O-glycosylation in snails does not display much variety. Four monosaccharide constituents – GalNAc, Gal, Man and Fuc – are the building blocks of the structures. The only further modification is a methylation of the hexoses, resulting in 3-*O*-Me-Gal, 4-*O*-Me-Gal and 3-*O*-Me-Man, which has already been shown for snail N-glycans [14–17, 23].

Each O-glycan contains only one amino sugar, which is the protein linked GalNAc. No other protein linked sugar has been detected. The linkage amino acid was not determined in detail, but due to the easy release by β-elimination the common GalNAc-Ser/Thr motif is very likely. The connection to other amino acids, for example tyrosine, is usually more stable [29]. In snail O-glycans the inner GalNAc is frequently elongated by two 4-*O*-Me-Gal residues in (1–3)- and (1–6)-linkage. Elongations of this trisaccharide core by one or two hexoses with or without methyl group appeared in most snail species, whereas further elongation or fucosylation are rare events.

Insects, one neighbor of mollusks in the phylogenetic tree, display also internal GalNAc elongated by one Gal in their O-glycan structures, but further elongations and/or branching are frequently done by another amino sugar (GalNAc or GlcNAc). Some insect glycans are modified by phosphoethanolamine but never by methylation [30]. The closest relation of snail glycans is seen to worms. The main O-glycans of *Toxocara canis* are trisaccharides consisting of GalNAc, Gal or 4-*O*-Gal and 2-*O*-Fuc [9].

So far nothing is known about biological functions of snail glycans. For sure these glycans are similarly involved in recognition processes as they are in all other species, but especially the significance of the high degree of methylation of hexoses of N- as well as of O-glycans is waiting for elucidation.

Here we enlarge the current knowledge on glycosylation abilities of lower animals by presenting the first O-glycan analysis of snails, representatives of the mollusk phylum.

## Abbreviations

EI: Electron impact
ESI: Electrospray ionisation
Fuc: Fucose
Gal: Galactose
GalNAc: *N*-acetylgalactosamine
GC: Gas chromatography
GlcNAc: *N*-acetylglucosamine
Hex: Hexose
HexNAc: *N*-acetylhexosamine
HPLC: High performance liquid chromatography
LC: Liquid chromatography
Man: Mannose
Me: Methyl
min: Minutes
MS: Mass spectrometry
MS/MS: Tandem mass spectrometry
PGC: Porous graphitized carbon
red: Reduced
